# Evaluating the Appropriateness of Pulmonary Embolism Admissions in a Community Hospital Emergency Department

**DOI:** 10.7759/cureus.24292

**Published:** 2022-04-19

**Authors:** Salam Brikho, Marc T Zughaib, Grace Tsaloff, Ken Smythe, Marcel E Zughaib

**Affiliations:** 1 Internal Medicine, Ascension Providence Hospital, Southfield, USA; 2 Internal Medicine, Michigan State University College of Human Medicine, Lansing, USA; 3 Cardiology, Ascension Providence Hospital, Southfield, USA

**Keywords:** risk stratification, hestia, pesi, quality improvement research, emergency medicine, cardiology, pulmonary embolism (pe)

## Abstract

Pulmonary embolism (PE) is a diagnosis on the broader spectrum of venous thromboembolic (VTE) disease. The diagnostic key for clinicians is detecting which patients have a “high risk” of complications or mortality and who are in the “low-risk” population. The Pulmonary Embolism Severity Index (PESI) and HESTIA scores are validated risk stratification tools to determine if patients diagnosed with PE can be successfully managed in the outpatient versus inpatient setting. We aimed to investigate the appropriateness of PE admissions to our institution based on the risk stratification recommendations from PESI and HESTIA scores.

We retrospectively identified 175 patients admitted with a diagnosis of PE over one year at our hospital. Baseline demographics, length of admission, and admitting diagnoses were collected for all patients included in this study. PESI and HESTIA scores were then calculated for all included patients.

The average PESI score was 91.65 (95% confidence interval: 86.33, 96.97). There were 87 patients (49.7%) that had a low or very low PESI score of fewer than 85 points. Fifty-seven patients (33.7%) presented with a HESTIA score of 0. The risk stratification score indicates these patients as low risk, and appropriate for outpatient management. However, they were instead admitted to the hospital which contributes to increased costs, risk of adverse events, etc. There were 0 mortalities reported for patients in the "low or very low risk" groups, with four reported mortalities in the “very high risk” groups.

In our cohort, 33.7%-49.7% of admissions for PE were risk-stratified as “low risk” and qualified for outpatient management based on HESTIA and PESI risk stratification scores, respectively. The underutilization of validated risk scores upon initial diagnosis of PE may lead to worse outcomes and increased healthcare expenditure.

## Introduction

Pulmonary embolism (PE) is a diagnosis in the broader spectrum of venous thromboembolic (VTE) disease, which is the third leading cause of cardiovascular death in the United States [1]. While the real incidence of VTE in the US population is unknown due to misdiagnosis and poor data collection, recent estimates suggest that as many as 900,000 people are affected in the US each year [2]. Mortality rates of PEs differ based on the severity of clot burden. For example, the mortality rate can range from 25% to 65% for patients with massive PE (a PE with hemodynamic instability) and less than 1% for patients with low-risk PEs and normal right ventricular systolic function started on anticoagulants [3]. 

Hospitalizations for PE increased from 23 per 100,000 people in 1993 to 65 per 100,000 people in 2012, according to Nationwide Inpatient Sample (NIS) data [4]. Additionally, adjusted mortality rates attributed to PE were 5.0, 3.4, and 3.5 per 100,000 people in 1999, 2008, and 2018, respectively [5]. From 2006 to 2010, >90% of all emergency department patients diagnosed with PE in the US were hospitalized [6].

The diagnostic key for clinicians is detecting which patients have a “high risk” of complications or mortality and who are in the “low-risk” population. Categorizing patients as “low-risk” with a potential for outpatient treatment can be met with resistance by practicing clinicians. This is mainly out of concern for potential complications. These concerns may explain the increase in hospital admissions for the diagnosis of PE.

The Pulmonary Embolism Severity Index (PESI) is a tool that was created in 2005 by Aujesky et al. to stratify risk in patients with PE [6]. The PESI score has since been verified extensively in multiple settings and is the preferred tool of the European Society of Cardiology to predict 30-day outcomes of patients diagnosed with a PE [6-14]. The PESI score is derived by a combination of demographics, comorbidities, vital signs, and clinical findings. These factors are weighted to provide an overall score from 0 to 125 (Appendix Table 3). Patients who have scores ≤ 65 indicate a very low risk of 30-day mortality (0.0%-1.6%). Scores of 66-85 indicate low risk (1.7%-3.5%). Scores of 86-105 indicate intermediate risk (3.2%-7.1%). Scores of 106-125 indicate high risk (4.0%-11.4%). And scores >125 indicate a very high risk (10%-24.5%) (Appendix Table 4).

The HESTIA Criteria are a set of exclusionary criteria for outpatient treatment created by Zondag et al. in 2011 that can be used alone or in conjunction with other risk stratification tools to determine the safety of possible outpatient treatment for patients diagnosed with PE [12]. Hestia criteria have also been validated in a number of settings [6,9]. HESTIA criteria assign one point to patients who have hemodynamic instability, need for thrombectomy, high bleeding risk, high oxygen requirements, severe pain requiring IV medications, a PE diagnosed on anticoagulation, creatinine clearance <30 mL/min, liver dysfunction, pregnancy, and presence of heparin-induced thrombocytopenia (HIT) (Appendix Table 5). A HESTIA score of 0 indicates a mortality risk of 0% and a VTE risk of 2%. A HESTIA score greater than 0 indicates the patient is "not low risk" and can meet the criteria for inpatient management [15-17] (Appendix Table 6).

While it has been shown outpatient treatment for low-risk patients with PE is safe and may benefit the patient both medically and financially, the concept continues to be met with significant resistance secondary to confounding social factors and clinician judgment. Our study aimed to investigate whether our hospital admissions of patients with PE were in line with the risk stratification recommendations based on PESI and HESTIA scores. This quality improvement study will allow us to identify and rectify potential areas of improvement that can be made in regard to guideline-directed management of PEs.

## Materials and methods

We retrospectively identified patients who were admitted with a diagnosis of PE from January to December 2019 at our facility. Initially, 588 patients were identified with admissions for PESI during 2019. We aimed to include a study sample of 175 patients, so we decided to systematically include every third patient from the overall population. This methodology would allow us to reduce selection bias while still obtaining a representative sample population. Inclusion criteria included patients older than 18 years old who were admitted to the hospital with a diagnosis of PE. Patients were excluded if they were admitted for a separate medical or social diagnosis, or if no PE was actually demonstrated on imaging.

Baseline demographics, length of admission, admitting diagnoses, vital signs, and laboratory results were collected for all patients included in this study. CT angiogram reports were reviewed for all included patients to confirm positive PE. PESI and HESTIA scores were then calculated for all included patients. Continuous variables were analyzed with Microsoft Excel.

## Results

We included 175 hospitalized patients in our study. The average age was 63.3 years old (95% confidence interval: 61.35, 65.91). There were 70 males (40%) and 105 females (60%). Ninety-eight (98) patients were Caucasian (56%), and 77 patients were African-American (44%). The average length of stay was 4.69 days (95% confidence interval: 4.06, 5.33) (Table 1).

**Table 1:** Baseline characteristics for the total population, n = 175.

	Mean/n	95% Confidence Interval
Age	63.3	(61.35, 65.91)
Gender		
- Male	70	
- Female	105	
Race		
- Black	77	
- White	98	
Length of Stay	4.69	(4.06, 5.33)

**Figure 1:**
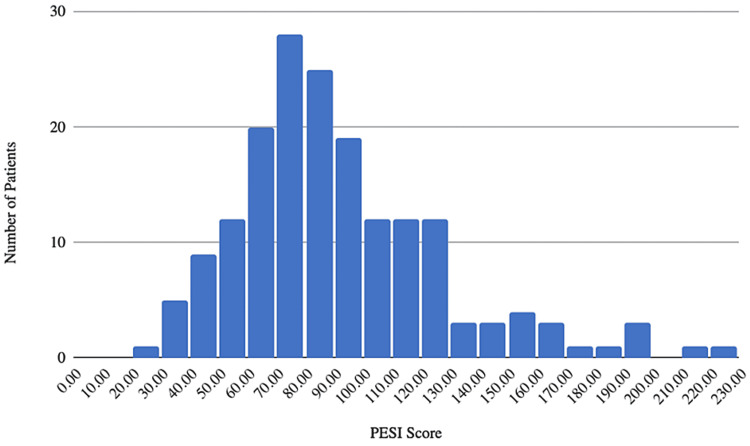
Distribution of PESI scores. PESI less than 85 indicates low risk and is appropriate for outpatient treatment. PESI - Pulmonary Embolism Severity Index

The average PESI score was 91.7 (95% confidence interval: 86.33, 96.97) (Table 2). There were 34 patients (19.4%) with a very low-risk PESI score of less than or equal to 65 points (Figure 1). There were 53 patients (30.3%) with a low-risk PESI score between 66 to 85. There were 87 patients (49.7%) that had a combined very low or low-risk PESI score of fewer than 85 points, 42 patients (24%) had an intermediate risk PESI score between 85 and 105 points, 20 patients (11.4%) had a high-risk PESI score between 106 and 125 points, and 26 patients (14.9%) had a very high-risk PESI score greater than 125 points.

**Table 2:** Patients with PESI scores (n=175) and HESTIA scores (n=169) that are considered low or very low risk and qualify for outpatient treatment of PE.

Average PESI score(95% CI)	91.7 (86.3, 97.0)
Patients with PESI < 85	87
% of PESI <85	49.7%
Patients with HESTIA of 0	57
Total percentage of patients with HESTIA of 0	33.7%

**Figure 2:**
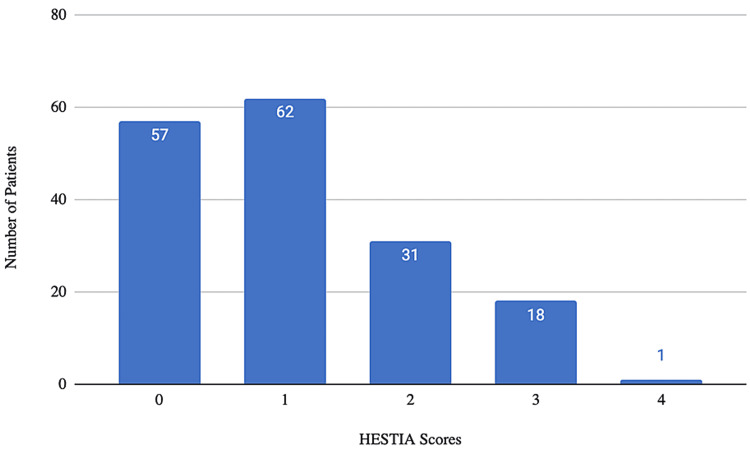
Number of patients within each HESTIA score category. HESTIA score <1 demonstrates a patient has a mortality risk of 0% and can be treated successfully in the outpatient setting (N=169).

Fifty-seven patients (33.7%) had a HESTIA score of 0 (Figure 2), 62 patients (37.3%) had a HESTIA score of 1, 31 patients (18.3%) had a HESTIA score of 2, 18 patients (10.7%) had a HESTIA score of 3, and one patient (0.01%) had a HESTIA score of 4. There were a total of 38 patients with very low or low PESI score with a HESTIA score of 0 (38/87, 43.7%). The remainder of patients with very low or low PESI scores (n=49) had a HESTIA score > 0.

There were 11 patients that were very low or low risk based on PESI scores that ultimately underwent intervention. Out of the 11 patients, seven were selected for intervention based on the PE Response Team's (PERT) decision. There were three patients taken for intervention based on oxygen desaturation below 92%, two patients that were triaged to the ICU, one patient with symptomatic bradycardia, and one patient with evidence of right heart strain on echocardiogram. There were zero patients with a HESTIA score of 0 that underwent intervention. There were no mortalities in patients that were low risk based on PESI or HESTIA criteria (0% mortality rate). Overall, the population had four mortalities, all of which were characterized as very high risk on PESI criteria (mortality rate 4.8%).

## Discussion

Studies have shown up to 50% of patients admitted for PE in the US could be safely treated in an outpatient setting [8]. Outpatient treatment of low-risk PE patients has been proven to be safe in terms of mortality, recurrent VTE, and major bleeding events [6-12]. Erkens et al. [10] found that in a cohort of 473 patients at Ottawa Hospital in Canada, there was no statistically significant difference in outcomes between the 260 people treated in the outpatient setting and the 213 people treated in the inpatient setting. Aujesky et al. [13] reached a similar conclusion with their cohort of 344 patients (171 inpatients, 173 outpatients) from the US, Belgium, and France who were diagnosed with low-risk PE. Another study performed by Roy et al. [11] found a statistically significant increase in major bleeding events, recurrent venous thromboembolism, and death among hemodynamically stable patients diagnosed with PE treated in an inpatient setting compared to matched cohorts treated as outpatients. Peacock et al. [6] determined that total patient costs are reduced by a median of $2,496 when outpatient treatment is pursued compared to standard of care treatment in the US. In fact, due to safety and reduced cost, current international guidelines from the European Society of Cardiology recommend outpatient anticoagulant treatment for low-risk patients [14,18]. 

In our study, 34 patients (19.4%) had a very low-risk PESI score and an additional 53 patients (30.3%) had a low-risk PESI score on admission. Combining the two low and very low-risk groups increases a total of 87 patients (49.7%) who had a PESI score less than 85. Using the PESI risk stratification, these 87 patients would have likely achieved successful management of their PE in the outpatient setting. Similarly, 57 patients (33.7%) in our cohort had a HESTIA score of 0. This score indicates these patients as low risk. These patients were also appropriate for outpatient management but were instead admitted to the hospital. Our rates were similar to a recent HOME-PE study presented at the European Society of Cardiology Virtual Congress in which 48.4% of the PESI group and 39.4% of the HESTIA group were eligible for outpatient care for their PEs [19].

There were 87 patients characterized as low or very low risk by PESI criteria, but 49 of these met one or more HESTIA criteria. As these 49 patients were admitted for various hemodynamic, social, and clinical reasons, a total of 11 patients ultimately underwent intervention. Interventions included thrombectomy, Ekosonic Endovascular System (EKOS), or embolectomy. There were zero mortalities for patients in the low-risk groups based on either PESI or HESTIA criteria. There were four mortalities in patients characterized as intermediate risk or higher on PESI criteria (4.8%). This information can further support the use of these scoring systems upon initial triage.

These data create an opportunity for improvement in our admission process for patients diagnosed with PEs. All patients diagnosed with PESI at our institution are discussed with the PERT to decide which patients can be managed medically or procedurally. The PERT includes interventional cardiologists, interventional radiologists, and vascular surgeons. However, the admission decision is ultimately up to our emergency department physicians. Increased efforts by physicians and administrators to provide education on these validated risk stratification scores could lead to improvements in decision-making. We acknowledge the caveats that no patient is defined by a single diagnosis, admission diagnosis can be a complicated aggregation of several factors, and eventually, the clinical decision acumen of the treating physician greatly influences the treatment course. Education about PESI and HESTIA scores and integration into admission decisions could lead to decreased admissions, improved patient outcomes, and a decrease in healthcare spending.

Our limitations include this investigation as a single-center study. Additionally, the retrospective use of some of the PESI and HESTIA criteria (including patient stability and mentation) is somewhat based on clinician judgment and can be subjective. As this was a retrospective study, we are unable to fully enrapture the medical decision-making process and conversations between the emergency department and PERT physicians. Clinical expertise and acumen are difficult to control for and may have played a role in the selection of low-risk patients to undergo intervention. Social determinants of health may play a role in the triage of these patients. These include family support, access to transportation, and access to outpatient treatment locations. Further research on whether community hospitals are aligning treatment settings with risk stratification is needed to explore possible areas of opportunity for improvement.

## Conclusions

Outpatient treatment for low-risk patients with PE has been shown to be safe and effective in several studies. However, this concept has not been fully adopted in the United States. In our retrospective study, between 33.7% and 49.7% of admissions for PE could have been risking stratified as “low risk” and qualified for outpatient management based on PESI and HESTIA risk stratification scores. The use of validated risk scores upon initial diagnosis of PE may lead to safer and more cost-effective treatment strategies. These risk scores can further be used to decrease patient mortality and better utilize healthcare resources.

## Appendices

**Table 3:** PESI independent predictors of 30-day mortality in the derivation sample and points assigned to the risk score. *Defined as disorientation, lethargy, stupor, or coma. †With and without the administration of supplemental oxygen. Adopted from Aujesky et al. [12].

Predictors	β-Coefficients (95% CI)	Points Assigned
Demographic characteristics		
Age, per yr	0.03 (0.02–0.03)	Age, in yr
Male sex	0.17 (0.02–0.32)	+10
Comorbid illnesses		
Cancer	0.87 (0.71–1.03)	+30
Heart failure	0.31 (0.14–0.49)	+10
Chronic lung disease	0.30 (0.12–0.47)	+10
Clinical findings		
Pulse ⩾ 110/min	0.60 (0.44–0.76)	+20
Systolic blood pressure < 100 mm Hg	0.86 (0.67–1.04)	+30
Respiratory rate ⩾ 30/min	0.41 (0.23–0.58)	+20
Temperature < 36°C	0.42 (0.25–0.59)	+20
Altered mental status*	1.50 (1.30–1.69)	+60
Arterial oxygen saturation < 90%†	0.58 (0.37–0.79)	+20

**Table 4:** PESI score risk assessment classes I-V.

PESI	Total score	30-day mortality
Class I	< 66 very low risk	0%-1.6%
Class II	66-85 Low risk	1.7%-3.5%
Class III	86-105 intermediate risk	3.2%-7.1%
Class IV	106-125 high risk	4%-11.4%
Class V	>125 very high risk	10%-24.5%

**Table 5:** HESTIA criteria for outpatient treatment of pulmonary embolism. Adopted from Zondag et al. [11].

HESTIA criteria
Is the patient hemodynamically unstable?
Is thrombolysis or embolectomy necessary?
Active bleeding or high risk of bleeding?
More than 24 hours of oxygen supply to maintain oxygen saturation > 90%?
Is pulmonary embolism diagnosed during the anticoagulant treatment?
Severe pain needed intravenous pain medication for more than 24 hours?
Medical or social reason for treatment in the hospital for more than 24 hours?
Does the patient have a creatinine clearance of < 30 mL/min?
Does the patient have severe liver impairment?
Is the patient pregnant?
Does the patient have a history of heparin induced thrombocytopenia?
If the answer to one of the questions is "yes," the patient cannot be treated at home in the Hestia Study

**Table 6:** HESTIA score risk assessment.

HESTIA score	Risk
0	0% mortality, 2% VTE recurrance
>0	Not low, needs inpatient
